# Ultrawide Field Imaging in Diabetic Retinopathy: Exploring the Role of Quantitative Metrics

**DOI:** 10.3390/jcm10153300

**Published:** 2021-07-27

**Authors:** Mohamed Ashraf, Jerry D. Cavallerano, Jennifer K. Sun, Paolo S. Silva, Lloyd Paul Aiello

**Affiliations:** 1 Beetham Eye Institute, Joslin Diabetes Center, Boston, MA 02215, USA; jerry.cavallerano@joslin.harvard.edu (J.D.C.); jennifer.sun@joslin.harvard.edu (J.K.S.); paoloantonio.silva@joslin.harvard.edu (P.S.S.); lloydpaul.aiello@joslin.harvard.edu (L.P.A.); 2 Ophthalmology Department, Alexandria Faculty of Medicine, Alexandria Governorate 21500, Egypt; 3 Ophthalmology, Harvard Medical School, Boston, MA 02215, USA

**Keywords:** diabetic retinopathy, ultrawide field imaging

## Abstract

Ultrawide field imaging (UWF) has allowed the visualization of a significantly greater area of the retina than previous standard approaches. In diabetic retinopathy (DR), significantly more lesions are seen on UWF imaging compared to the seven-standard ETDRS fields. In addition, some eyes have lesions that are located predominantly in the peripheral retina that are associated with an increased risk of DR progression. The current DR severity scales are still largely based on clinically visible retinal microvascular lesions and do not incorporate retinal periphery, neuroretinal, or pathophysiologic changes. Thus, current scales are not well suited for documenting progression or regression in eyes with very early or advanced DR, nor in the setting of vascular endothelial growth factor inhibitors (antiVEGF). In addition, the categorical system is highly subjective, and grading is variable between different graders based on experience level and training background. Recently, there have been efforts to quantify DR lesions on UWF imaging in an attempt to generate objective metrics for classification, disease prognostication and prediction of treatment response. The purpose of this review is to examine current quantitative metrics derived from UWF fluorescein angiograms and UWF color imaging to determine their feasibility in any potential future DR classification.

## 1. Introduction

**Figure 1:**
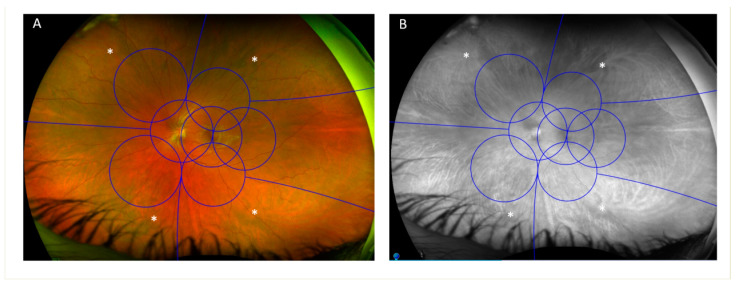
Early treatment diabetic retinopathy study (ETDRS) seven-standard fields compared to ultrawide fields (UWF). (**A**) Color UWF image showing an overlay of the seven scheme fields. (**B**) A green-filtered image of the same eye showing enhanced visibility of the choroidal vessels and locations of the vortex veins (white asterisk).

In 2019, an expert panel defined ultrawide field imaging (UWF) as images showing retinal anatomic features anterior to the vortex vein ampullae in all four quadrants [1]. UWF imaging allows the visualization of a substantially greater area of the retina compared to the standard seven field Early Treatment Diabetic Retinopathy Study (ETDRS) fields (82% vs. 30%) (Figure 1) [2,3]. For diabetic retinopathy (DR), grading UWF images has substantial agreement (K = 0.77–0.84) with ETDRS standard photos as demonstrated in cross-sectional, multi-center studies [2,4,5,6]. Furthermore, UWF allows the identification of DR lesions predominantly outside the ETDRS seven-standard fields, referred to as predominantly peripheral lesions (PPL). Several studies have demonstrated that PPL are present in 30–40% of eyes with DR [2,6,7]. A recent DRCR retina network (DRCR.net) study reported that the presence of PPL suggested a more severe DR level in 11% of eyes, with hemorrhages/microaneurysms (H/Ma) being the predominant peripheral lesion, thereby confirming previous work by Silva et al [2,6,8]. In addition to their value in DR grading, PPL have been associated with a 3.2 fold increased risk of two-step or more DR progression (11% vs. 34%, *p* = 0.005) and a 4.7 fold increased risk for progression to proliferative DR (6% vs. 25%, *p* = 0.005) over four years [8].

The use of accurate methods to classify DR that are reflective of its natural history is critical for studies on pathogenesis and treatment of diabetic retinal disease. The landmark clinical trials that have described and characterized DR have used the modified Airlie House Classification Scale proposed in the ETDRS. ETDRS severity levels have been used to guide clinical practice recommendations for follow-up interval and treatment initiation. The ETDRS scale defines 13 eye levels and 26 patient levels of DR severity, and has been used extensively in research and clinical trial settings [9]. However, the American Academy of Ophthalmology formed a consensus panel in 2003 to create a simplified classification called the International Clinical Diabetic Retinopathy (ICDR) severity scale [10]. This five-step scale for DR combined ETDRS levels and used simplified descriptions of categories to promote greater adoption in routine clinical practice. However, the ICDR does not replace ETDRS levels of DR in large-scale clinical trials or studies in which precise DR classification is necessary. Both ETDRS and ICDR severity scales provide guidance for treatment but remain only broad estimates of clinical outcomes for individual eyes and cannot fully predict the course of the DR for specific patients.

Although the ETDRS and ICDR severity scales are stepped scales with defined categories, neither is quantitative and the estimates for increased rates of progression with increased DR severity level do not increase in a linear fashion. The current DR severity scales are still largely based on clinically visible retinal microvascular lesions and do not incorporate retinal periphery, neuroretinal, or pathophysiologic findings in early non-clinically evident DR, nor visual function. Thus, the current scales are not well suited to document progression or regression in eyes with very early or advanced DR or in the setting of vascular endothelial growth factor inhibition (antiVEGF) [11].

Recently, it has been suggested that a generally inclusive, multidimensional severity scale should be developed to more accurately characterize DR and the risk of progression [11]. This proposed scale might incorporate information from complex approaches and novel features on retinal images that are not detectable by human grading. One possible addition to this classification could include quantitative metrics derived from UWF imaging, whether derived from UWF fluorescein angiography (FA) or UWF color images (CI). There are other UWF imaging techniques such as fundus autofluorescence (FAF) and indocyanine green angiography (ICGA); however, current literature is limited regarding their use of quantitative metrics in eyes with DR. In addition, the purpose of this review is to examine current quantitative metrics derived from only UWF-FA and UWF-CI to determine their feasibility in any potential future DR classification. The current review does not explore how other possible diseases or pathologies (retinal vein occlusion, hypertension, hematologic malignancies, etc.) may confound the diagnosis or grading of retinopathy [12].

## 2. Hemorrhages/Microaneurysms

Hemorrhages and microaneurysms (H/Ma) are among the earliest clinical signs of DR and their presence is central to the diagnosis of any DR severity level [13]. The severity and extent of H/Ma have been used to grade DR and indirectly determine the risk of progression [9]. Previous studies looking at central retinal fields did not identify a robust association between H/Ma counts and DR progression [14,15]. It is still unknown if incorporating H/Ma counts from UWF images can improve prediction and determine which eyes are at increased risk of developing proliferative diabetic retinopathy (PDR) or diabetic macular edema (DME).

While manual counting has been employed in many previous multicenter studies and clinical trials, the counting was usually limited to the use of FA and the evaluation of central macular fields [14,15,16,17,18]. Manual counting can be immensely laborious and time consuming and only a few studies have attempted to quantify H/Ma counts on UWF color images (UWF-CI) [19,20,21,22]. Quantitative H/Ma counts determined that on average UWF images identified 21.3 more H/Ma in the peripheral extended fields compared to the ETDRS fields, representing an approximate 50% increase in the total counts per eye [22]. Another study by Sadda et al. using manual H/Ma counts determined that eyes with greater H/Ma counts and wider H/Ma distribution were more likely to progress to PDR [21]. Only one study performed manual microaneurysm counts on UWF-FA and determined that compared to CI, UWF-FA microaneurysm counts were 4.5 fold higher overall, 3.2 fold higher in the ETDRS fields and 5.3 fold higher in the extended ETDRS fields [19]. Using only microaneurysm counts to grade DR, agreement rates between UWF-FA and UWF-CI DR grades were moderate whether ETDRS fields (k = 0.346) or UWFs (ETDRS fields + extended fields) (k = 0.317) were used. This agreement rate improved substantially after dividing total counts by three for ETDRS fields and four for UWF (k = 0.600 and k = 0.565, respectively). Quantitative H/Ma counts can also be used to grade PPL and has previously been shown to have a high agreement rate with qualitative subjective grading (96%, k = 0.858) [23].

There have also been attempts at automated microaneurysm quantification on UWF-FA images [24,25]. Perhaps the most widely adopted method was that described by Ehlers et al., which performed well against manual grading and had high repeatability between early phase and late phase angiogram counts [26]. On a data set of 304 eyes with DR with and without DME, it was reported that eyes with DME had significantly greater posterior pole microaneurysm counts compared to those with no DME (69.6 ± 54.2 vs. 49.7 ± 51.3, *p* = 0.003) and that increasing counts were an independent risk factor for the presence of edema [27]. Furthermore, in a study of 339 eyes with all levels of DR severity, increased panretinal automated microaneurysm counts on UWF-FA were associated with increased DR severity, findings that are similar to the study by Ashraf et al. using manual counts [19,28]. In a post hoc analysis of the RECOVERY study, eyes with PDR treated with aflibercept were found to have decreased automated UWF-FA microaneurysm counts at six months and one year in both the Q4 and Q12 groups [29].

Automated H/Ma counts on UWF-CI have also been attempted [30,31]. The automated tool by Optos^®^ (Optos, Denfermline, UK) is perhaps the more widely studied of the two available methods. Using their diabetic lesion detection tool (DLD), H/Ma were automatically quantified in each of the individual ETDRS fields and their respective extended fields [31]. PPL was then graded in each image using those counts and PPL-H/Ma was considered present if the total count in the extended field exceeded that of its corresponding ETDRS field. Using the automated algorithm on a set of 1712 eyes, 20% of eyes were identified as having PPL, and the risk of DR progression was two to three times greater in eyes with PPL than in those without PPL. The automated tool was also employed on a dataset of 13,015 eyes and identified an association between renal function, anemia, and the presence of PPL [32].

Work by our group looking at parameters affecting PPL grading determined that agreement between quantitative methods and qualitative methods was moderate (ĸ = 0.423, *p* < 0.001) overall with fair agreement in eyes with mild nonproliferative diabetic retinopathy (NPDR) (ĸ = 0.336, *p* < 0.001) and moderate agreement in eyes with moderate NPDR (ĸ = 0.525, *p* < 0.001) and severe NPDR-PDR (ĸ = 0.409, *p* < 0.001) [33]. Increasing the threshold for quantative PPL grading improved the agreement rates between the methods, with peak agreements at H/Ma count differences of six for mild NPDR, five for moderate NPDR and nine for severe NPDR—PDR. Futhermore, there were differences in agreement rates between qualitative and quantitative methods based on the UWF imaging device type (Optos California vs. Optos 200 TX), with the 200 TX having very poor agreement in eyes with mild NPDR. Therefore, it was concluded that the determination of PPL depends on use of qualitative or quantative methods, DR severity, device type, and magnitude of lesion differences used for quantative assessment. It will be important in future studies to determine which of these methods for quantifying PPL is more accurate and, more importantly, which is more highly associated with future DR progression in a prospective, longitudinal cohort.

## 3. Visible Retinal Area

Given the wide variability in the total visible retinal area (VRA, mm^2^) on UWF imaging, a study was conducted to evaluate whether manual lid retraction and pupillary dilation affect VRA, automated H/Ma quantification and automated PPL-H/Ma grading [34]. The study included 5919 eyes and found that manual lid retraction increased VRA by 10% and increased the mean number of H/Ma detected on UWF-CI by 41.7%. Manual lid retraction in turn resulted in increased detection of PPL, whether by human graders or using an automated technique. Thus, given the importance of H/Ma counts and PPL in determining the risk for DR progression, teleophthalmology programs and clinical trials should attempt to maximize VRA for optimal risk assessment.

## 4. Arteriolar and Venular Diameters

Retinal vascular caliber has been studied in several large multi-center and population-based studies as possibly being associated with DR development and progression [35]. These studies measured vascular calibers in an area immediately adjacent to the optic disc and provided summary indices—central retinal artery equivalent (CRAE) and central retinal vein equivalent (CRVE)—which provided an estimate of overall vascular changes in the entire retina.

A recent study using UWF-CI explored the association of retinal nonperfusion index (NPI) and DR severity with the location of vascular caliber measurements [36]. Using a previously validated semi-automated software (OptomapAVR, Optos plc, Dunfermline, UK), measurements were done in two zones: an inner zone 1–1.5 optic disc diameters (DD) from the optic disc center which represented more central vascular calibers and an outer zone 3.25–4.25 DD from the optic disc center representing more peripheral vasculature. Although there were no associations between inner arteriolar diameters and retinal nonperfusion, in the outer zone eyes with the thinnest arteriolar calibers were associated with a 1.7–2.4 fold increase in nonperfusion across different retinal zones (posterior pole, mid-periphery and far-periphery). Venular calibers (inner and outer) were not associated with nonperfusion regardless of the retinal zone. Finally, in eyes with versus without PPL, thinner outer zone retinal arteriolar caliber was more common (34.1% vs. 20.8%, *p* = 0.017) as well as thicker outer venular caliber (33.0% vs. 21.3%; *p* = 0.036) in those with PPL. The study concluded that peripheral arteriolar narrowing was associated with increased nonperfusion and presence of PPL, highlighting the importance of measuring vascular calibers in the peripheral retina using UWF imaging.

## 5. Retinal Nonperfusion

Several studies have divided the retina into three zones using two pre-specified concentric rings centered on the fovea [24,37,38]. The posterior zone was defined as the retinal area within a 10 mm radius circle centered on the fovea, the mid-periphery as the area between a 10 mm and 15 mm radius circle and the far-periphery the area beyond the 15 mm radius circle. Nonperfusion can be quantitatively determined in those zones in eyes with retinal vascular diseases such as DR using UWF-FA. Using a standardized manual technique, Silva et al. demonstrated that trained graders have a high intragrader (r = 0.95) and intergrader (r = 0.86) agreement for nonperfusion measurements [23].

Nonperfusion measurements using UWF-FA in eyes with DR have allowed a more detailed characterization of ischemia in eyes with DR and their role in treatment response. Nonperfusion increased with increasing DR severity in all retinal zones [23,28]. There was also an association between the presence of PPL and increased peripheral nonperfusion. In eyes with PPL, fields having PPL were associated with 33–60% more nonperfusion compared to a corresponding field without PPL [23]. Results from the DAVE study and another study by Talks et al. confirmed that the addition of targeted laser to areas of nonperfusion in patients with anti-VEGF resistant diabetic macular edema (DME) did not reduce the treatment burden or improve outcomes [39,40].

The quantification of nonperfusion in DR is important given its possible association with DR progression. While the ETDRS did not find substantive additional prognostic value in FA metrics of the macula, it is unknown if UWF-FA metrics may provide additional value [41,42]. A recent study identified a threshold for total nonperfusion area (77.48 mm^2^) above which patients were at increased risk of developing PDR [43]. This threshold had limited sensitivity and specificity (59.5% and 73.6%) with an AUC of 0.7. The study also did not identify individual thresholds for distinct retinal zones. Nicholson et al. used eyes from the CLARITY study and reported significant differences in nonperfusion area between eyes with severe NPDR and PDR in the periphery but not the posterior pole [44]. The study did not divide the peripheral retina into the mid-periphery and far-periphery. It also used a different method for quantifying nonperfusion. Using a threshold of 118.3 disc-area total nonperfusion area, the authors reported a sensitivity of 84.9% and specificity of 66.1% to identify PDR. These studies did not stratify eyes into those with and without predominantly peripheral lesions (PPL), although these eyes likely represent a distinct group with differences in nonperfusion distribution and risk for DR progression [6,8,23]. The RECOVERY study looking at a cohort of eyes with PDR reported that eyes with retinal neovascularization elsewhere (NVE) had a significantly higher NPI in the mid-periphery [45]. The study failed to find an association between NVE location in the mid-periphery and far-periphery and increased NPI in the same retinal zones. With regards to neovascularization of the disc (NVD), NPI was only increased in the mid-periphery but not the posterior pole or far-periphery.

Although it is unclear if the vortex veins truly represent an anatomic transition point from a bilaminar to unilaminar vascular arrangement, the location of the vortex veins has traditionally been used to demarcate the boundary between the retinal mid and far periphery. While initially it was suggested that a 15 mm radius circle centered on the fovea represented the anatomic location of the vortex veins, recent UWF indocyanine green angiography (ICGA) studies have demonstrated that the actual location is closer to a 14 mm radius circle centered on the optic nerve [46]. A study by Rageh et al. demonstrated that circles with a radius of 15 mm centered on the optic disc are more anatomically accurate than those centered on the fovea at defining the transition from bilaminar to unilaminar vascular plexus. Centration of the circle on the optic disc rather than fovea significantly altered nonperfusion measurements in eyes with moderate NPDR in both the mid-periphery and far-periphery.

Many of the studies quantifying nonperfusion on UWF-FA have used non-steered on-axis images [23,36,43,44]. An important finding in the study by Rageh et al. was that nasal, superior, and inferior steering may be needed for more accurate quantification of NPI given the limited visibility of far peripheral retina on non-steered images [47]. Temporal steering is not needed since the vascular retina is completely visualized all the way to avascular retina in most eyes. Therefore, studies evaluating far peripheral nonperfusion and diabetic lesions should take into consideration the reduced ability to fully visualize the far peripheral retina in these quadrants.

## 6. Retinal Vascular Bed Area

Retinal vascular bed area (RVBA) is used to calculate the area and density occupied by the vessels on UWF-FA. RVBA is akin to vessel density metrics calculated on optical coherence tomography angiography (OCTA). It is calculated by binarizing UWF-FA images and the actual area of the vessels calculated depending on the location of the pixel on the image after projecting it back onto a sphere [48]. RVBA was found to be increased in eyes with worsening DR severity, being higher in those with PDR compared to those with NPDR [49]. However, this metric was poorly associated with nonperfusion measurements. Increased RVBA was also associated with the presence or absence of DME [38]. It was suggested that this novel metric might be more strongly compared to traditional nonperfusion measurements with DR severity. However, it is unclear whether RVBA can predict progression or worsening retinopathy.

## 7. Wide Field Optical Coherence Tomography Angiography (WF-OCTA) Quantitative Metrics

OCTA allows the noninvasive 3-D visualization of the different retinal vascular plexuses without the risks of injecting fluorescein dye [50,51]. There has been extensive work looking at OCTA quantitative metrics in patients with DR, primarily focused on the macular area (central 3 × 3 mm scans) [52,53]. Recently, wide field swept-source OCTA (WF SS-OCTA) has been evaluated in patients with DR and its utility is being compared to standard UWF-FA and UWF-CI.

### 7.1. Macular OCTA (3 × 3 mm)

The association of vascular density (VD) in the central macular area using 3 × 3 mm scans and UWF-CI has been evaluated [54]. The vascular layers were segmented into three plexuses; the superficial (SCP), intermediate (ICP) and deep capillary plexus (DCP) using optical coherence tomography angiography (OCTA) in eyes graded for DR severity on UWF-CI as well as the presence or absence of PPL. The study demonstrated that although VD decreased significantly in both the SCP and DCP with increasing DR severity in eyes without PPL, in eyes with PPL there were no significant differences in any of the vascular layers. The study also annotated UWF-CI for both H/Ma and intraretinal microvascular abnormalities (IRMA) and noted that counts for both lesions were negatively correlated with macular VD in eyes without PPL, but were not correlated with macular VD in eyes with PPL. These results support the hypothesis that central VD is correlated with DR severity only in eyes without PPL and suggest a need to stratify eyes based on PPL status prior to inclusion in any clinical trial evaluating OCTA metrics and DR.

Another study looked at the association of macular OCTA metrics with two UWF-FA quantitative metrics, microaneurysm counts and nonperfusion index (NPI) [55]. The study demonstrated that both UWF-FA metrics were more strongly associated with DR severity compared to SCP and DCP VD. Furthermore, there was only a moderate correlation between quantitative macular OCTA VD and UWF-FA metrics, regardless of the retinal zone being analyzed (posterior pole, mid-periphery or far-periphery).

### 7.2. Widefield OCTA (WF-OCTA)

WF-OCTA has expanded the field of view from 3 × 3 mm to 15 × 9 mm scans using the montage feature of the PLEX Elite 9000 (Carl Zeiss Meditec AG, Jena, Germany) [56]. The increased area represents approximately 80 degrees of the total retina, which is still significantly less than the 200 degrees visualized by UWF-CI and UWF-FA imaging. However, despite the smaller area, WF-OCTA can detect over 95% of all neovascularization (NV) detected on UWF-FA [57,58,59,60,61]. WF-OCTA also has the added advantage of using the vitreoretinal interface (VRI) angio slab to improve detection rates of neovascularization [62]. Similar rates of NV detection were reported using WF-OCTA and UWF-FA, regardless of the level of training for the grader (resident, retina fellow or attending) [63].

Areas of nonperfusion detection on UWF-FA co-localized with areas of nonperfusion on WF-OCTA, although the latter detected significantly greater ischemia [64,65]. Given that WF-OCTA accurately demonstrates the vascular network in neovascularization without the effect of leakage, quantifying the size of the NVE or NVD may prove to be a prognostic factor for the risk of vitreous hemorrhage development [66]. Furthermore, the VRI slab may facilitate the identification of NV that have traversed the posterior hyaloid and are at risk of causing vitreous hemorrhage [66].

## 8. Artificial Intelligence

While artificial intelligence (AI) has been studied extensively in the diagnosis of DR using images acquired of the posterior pole, there is limited data regarding its implementation on UWF images [67,68]. To date, there have only been a handful of papers tackling the role of deep learning algorithms in identifying DR on UWF images [69,70]. The largest study to date was that by Tang et al., which used 9392 images of 1903 eyes from 1022 subjects with diabetes. The study used a primary data set from Hong Kong and used four external validation data sets, one each from the UK and Argentina, and two from India. Images were labelled as to the presence of referable DR (RDR), defined as moderate NPDR or worse or if DME was detected, and vision threatening DR (VTDR), defined as severe NPDR or worse or if DME was detected.

For detecting RDR and VTDR, the area under the receiver operating characteristic curve (AUROCs) was 0.981 and 0.966, respectively, with a sensitivity/specificity of 94.9%/95.1% and 87.2%/95.8% for the primary data set. For the validation sets, the AUROC and accuracies for detecting RDR and VTDR were >0.9% and >80% respectively. In the future, AI techniques that evaluate a wider area of the retina with UWF imaging and incorporate neural, functional, and systemic quantitative parameters may have the potential to more accurately predict DR progression in individual eyes based on patient-specific characteristics.

## 9. Conclusions

Both UWF-FA and UWF-CI are potentially rich sources of novel quantitative metrics that may help better classify diabetic retinal disease and more accurately predict the risk of progression and visual loss. The most encouraging results so far from UWF investigations have involved H/Ma counts and nonperfusion. H/Ma counts correlate well with DR severity levels, and their quantification on UWF may be used in the future to improve prediction of disease progression. In addition, they may be used in the future as an objective quantitative determinant of treatment response to anti-VEGF. Increasing NPA on UWF-FA is also associated with more severe levels of retinopathy and the presence of neovascularization. Its clinical utility, however, has yet to be determined, with more longitudinal studies needed to evaluate whether it can better predict which eyes are at greater risk of DR progression independent of baseline ETDRS severity. The presence of PPL can also be determined from UWF quantitative metrics, but it will be important to define how quantitative PPL grading compares with qualitative grading with regards to predicting disease progression. With the use of quantitative metrics and AI approaches, advances in retinal imaging and image analysis have the potential to go beyond the visual evaluation of clinically evident retinal lesions. Future prospective, longitudinal studies in diverse patient cohorts throughout the natural history of the disease and compared with treatment exposure are needed to validate these promising UWF metrics as predictive or prognostic biomarkers in the diabetic eye.
